# Prenatal and Postnatal Choline Supplementation in Fetal Alcohol Spectrum Disorder

**DOI:** 10.3390/nu14030688

**Published:** 2022-02-06

**Authors:** Abigail M. Ernst, Blake A. Gimbel, Erik de Water, Judith K. Eckerle, Joshua P. Radke, Michael K. Georgieff, Jeffrey R. Wozniak

**Affiliations:** 1 Department of Psychiatry, University of Minnesota, Twin Cities, MN 55414, USA; ernst260@umn.edu (A.M.E.); ecke0056@umn.edu (J.K.E.); georg001@umn.edu (M.K.G.); 2 Proof Alliance, St. Paul, MN 55104, USA; gimbe013@umn.edu; 3 Great Lakes Neurobehavioral Center, Edina, MN 55435, USA; erikdewater@gmail.com; 4 Fagron Holdings, LLC, St. Paul, MN 55120, USA; joshua.radke@fagron.com

**Keywords:** fetal alcohol spectrum disorders, choline, brain, randomized controlled trials, longitudinal studies

## Abstract

Fetal alcohol spectrum disorder (FASD) is common and represents a significant public health burden, yet very few interventions have been tested in FASD. Cognitive deficits are core features of FASD, ranging from broad intellectual impairment to selective problems in attention, executive functioning, memory, visual–perceptual/motor skills, social cognition, and academics. One potential intervention for the cognitive impairments associated with FASD is the essential nutrient choline, which is known to have numerous direct effects on brain and cognition in both typical and atypical development. We provide a summary of the literature supporting the use of choline as a neurodevelopmental intervention in those affected by prenatal alcohol. We first discuss how alcohol interferes with normal brain development. We then provide a comprehensive overview of the nutrient choline and discuss its role in typical brain development and its application in the optimization of brain development following early insult. Next, we review the preclinical literature that provides evidence of choline’s potential as an intervention following alcohol exposure. Then, we review a handful of existing human studies of choline supplementation in FASD. Lastly, we conclude with a review of practical considerations in choline supplementation, including dose, formulation, and feasibility in children.

## 1. Introduction

Fetal alcohol spectrum disorders (FASD) represent a set of lifelong neurodevelopmental conditions resulting from prenatal alcohol exposure (PAE). FASD is a heterogeneous condition characterized by neurological abnormalities, cognitive and behavioral impairment, growth retardation, and craniofacial anomalies [1]. Recent estimates have suggested a global prevalence of 0.8%, and rates have been found to vary by region. FASD affects approximately 2.0 to 5.0% of the European and North American populations [2,3] and 13.6 to 28% of high-risk rural populations in South Africa [4,5]. Rates of alcohol consumption around the globe are generally increasing [6], with even larger accelerations seen during the recent COVID-19 pandemic [7]. Despite the tremendous public health burden it represents [8,9], FASD remains under-recognized and is often misdiagnosed [10,11]. FASD is an “umbrella” term, encompassing a number of subtypes, including fetal alcohol syndrome (FAS), partial fetal alcohol syndrome (PFAS), and alcohol-related neurodevelopmental disorder (ARND). FASD diagnoses are made on the basis of facial features (i.e., small palpebral fissures, smooth philtrum, smaller upper lip), growth deficits, neurological abnormalities, and neurocognitive/neurobehavioral deficits [1,12,13]. Neurocognitive deficits are a core feature of FASD, ranging from broad intellectual impairment to selective deficits in attention, executive functioning, memory, visual–perceptual/motor skills, social cognition, and academics [14,15]. Individuals with FASD are at an elevated risk of sensory processing abnormalities (e.g., hearing loss [16]), non-specific neurological deficits (e.g., cranial nerve abnormalities, ataxia, dysarthria [17,18]), and a wide range of comorbid conditions, including congenital malformations, chromosomal abnormalities, and behavioral disorders [19,20]. Importantly, these deficits persist into adulthood, and individuals with FASD can experience lifelong cognitive and physical impairment, psychiatric comorbidity, incarceration, homelessness, and other psychosocial challenges [9,21]. Although the literature on effective treatments for FASD has been growing, at present, very few interventions exist specifically for this population [22,23].

Cognition is a natural target for intervention in FASD because cognitive deficits contribute to real-world problems with adaptive functioning, social skills, and independent living [24]. One potential intervention for the cognitive impairments associated with FASD is the essential nutrient choline [25], which is known to have multiple direct effects on brain development and cognition in both typical and atypical development [26]. Here, we provide a summary of the limited but growing literature supporting the use of choline as a neurodevelopmental intervention for cognition and brain development in those affected by PAE. We first present an overview of PAE and FASD with an emphasis on alcohol’s potential to alter brain development, resulting in permanent cognitive impairments and functional deficits. We then provide a comprehensive overview of the nutrient choline and what is known about its role in typical brain development and its potential application in the optimization of brain development following early insult. Next, we outline the preclinical models that provide evidence of choline’s potential as an intervention following PAE. Then, we review the handful of existing human studies of choline supplementation following PAE. Lastly, we conclude the paper with a review of practical considerations in choline supplementation, including dose, formulation, and feasibility in children.

## 2. Alcohol’s Effects on the Developing Brain and Cognition

A wide range of human and animal studies have documented effects of PAE on brain structure and function across development [14,17,27,28]. In this section, we briefly summarize insights from the human and animal literature regarding mechanisms of alcohol teratogenesis and effects of PAE on neurodevelopment and related cognitive dysfunction.

### 2.1. Mechanisms of Alcohol Teratogenesis

Alcohol passes into the placenta and readily crosses the blood–brain barrier, directly affecting the developing fetus [28]. Multiple mechanisms by which alcohol affects the developing brain have been documented. There are known epigenetic effects, including effects on gene expression [29,30], such as DNA methylation and histone modification [31]. Excess neuronal apoptosis (i.e., programmed cell death) is a frequent finding following PAE, and oxidative stress and withdrawal-induced neuronal toxicity have also been described [29,30]. A growing body of evidence suggests PAE disrupts glial cell function and synaptogenesis [32]. Altered neuronal and glial cell proliferation and migration [29,30,32], including disrupted oligodendrocyte maturation, are thought to contribute to dysfunctional brain architecture and connectivity in PAE-affected organisms. In addition, PAE is known to impact placental health [33], to affect iron regulation in the developing fetal brain [34], and to disrupt maternal–fetal iron homeostasis [35], which may, in turn, contribute to neurodevelopmental abnormalities in areas such as the hippocampus. Notably, iron deficiency affects gray matter development, including hippocampal integrity and dendrite complexity [36,37], as well as white matter development, such as myelination [38].

### 2.2. Global Structural Abnormalities in Brain following PAE

Cross-sectional studies have documented a wide range of global neurological abnormalities linked to PAE (e.g., reduced total brain volume and/or reduced cerebral or cerebellar volumes at the group level), as well as regional structural abnormalities (e.g., corpus callosum shape distortions/thinning, gray and white matter abnormalities in frontal, parietal, and temporal lobes; volumetric reductions in the hippocampus, basal ganglia, thalamus; for comprehensive reviews, see Lebel et al. [39] and Moore et al. [40]). Recent studies using longitudinal designs have revealed differences in both gray and white matter developmental trajectories [41,42,43], abnormal cortical folding (i.e., gyrification; [44,45]), impaired functional connectivity [46,47], and altered brain–behavior relationships [43,48]. Importantly, these abnormalities are linked to the timing, frequency, and amount of alcohol exposure, genetics, and other prenatal and postnatal factors [49].

These neurodevelopmental effects contribute to the cognitive deficits consistently seen in individuals with FASD, which can range from profound and global deficits to more narrow impairments in specific cognitive domains, such as attention/executive functioning, learning and memory, visual-spatial skills, and motor skills [14,15]. General intelligence (i.e., IQ) is variable across affected individuals and across subtypes of FASD. The mean IQ for individuals with FASD as a group has been estimated to be as low as 72 (where the normative mean is 100, and the standard deviation is 15 [50]). Measured intelligence is lowest for individuals diagnosed with FAS, followed by those with the milder forms of FASD: PFAS and ARND [51]. Deficits in balance, motor coordination, fine motor control, and visual–motor integration [14,52], as well as language impairment in both expressive and receptive domains, have also been reported in individuals with PAE [53,54]. Social communication, behavior regulation, and adaptive skills are also commonly impaired [55,56]. Attention and executive function deficits are hallmark cognitive features of PAE [50,57]. Specific deficits have been reported in visual attention and aspects of shifting/orienting attention [46]. Executive function is often globally impaired with specific deficits in domains of set shifting/flexibility, concept formation/problem solving, planning, working memory, and inhibitory control, particularly on tasks involving complex information processing [50,58]. Notably, attention deficit/hyperactivity disorder (ADHD) is highly comorbid with FASD, and individuals with PAE and ADHD may demonstrate unique cognitive profiles and neurological abnormalities [59].

### 2.3. PAE May Disproportionately Affect the Hippocampus and Memory

The hippocampus has long been suspected to be particularly impacted by PAE, given the learning and memory deficits often observed in human and animal studies [58,60,61]. The hippocampus differentiates rapidly during fetal development, which may contribute to its particular vulnerability to insult in PAE. Ho et al. [62] first demonstrated that alcohol damages the hippocampus (and other subcortical structures) in animal models. Rodent behavior studies and parallel human studies of memory added to the understanding of hippocampal involvement in PAE [63,64]. Mechanisms by which PAE damages the hippocampus have also been investigated. Early reports indicated lower pyramidal neuron counts in the dorsal hippocampus in adult rats prenatally exposed to alcohol [65]. Subsequent studies have consistently demonstrated decreased cell counts and/or low dendritic density in CA1 of the hippocampus [66,67,68,69,70,71]. In addition to CA1, Livy et al. [67] reported decreased cell counts in CA3 and the granule cells of the dentate gyrus after gestational and early postnatal (third-trimester equivalent) alcohol exposure. Wigal et al. [71] reported an 11 percent reduction in granule cells in the dentate gyrus after gestational exposure, though this has not been consistently found [70,72,73]. PAE can also disrupt neurotrophin pathways and impair neurogenesis in the hippocampus into adulthood [74].

Although the extent of similar neuropathological findings in humans is unknown [75], recent advancements in MRI and automated processing methods have facilitated detailed morphology studies in humans. The hippocampus, along with other subcortical structures, including the amygdala and caudate, have been reported to be significantly smaller in children with PAE [76,77], although several studies have failed to find volumetric differences in these structures after correction for total brain volume [42,78,79,80]. A recent neuroimaging study examined hippocampal subfields and found significant volumetric reductions in 5 out of 10 regions in children with PAE compared to controls [60]. Importantly, the hippocampus is heavily interconnected with other systems [81,82,83], and its integrity is critical to the development, functioning, and organization of other domains. Together, neuroimaging studies have shown that PAE affects the structure, function, and connectivity of the hippocampus [60] and connected brain areas, including the prefrontal and parietal cortex [42,55,84], amygdala [42,76,85], and basal ganglia [76,86], suggesting that hippocampal-dependent learning and memory may be a particularly important domain for intervention.

### 2.4. White Matter and Network Connectivity

White matter abnormalities have been frequently found in both animal and human studies of prenatal alcohol exposure, reflecting significant volumetric reductions in important white matter pathways attributed to disrupted myelination [39,40,87]. The corpus callosum has been shown to be particularly susceptible to PAE-induced damage, with many studies reporting reduced volume and shape abnormalities, particularly in posterior regions [49,88,89]. Using diffusion tensor imaging (DTI) methodology, reduced white matter microstructural integrity has been consistently found in the corpus callosum, cingulum, cerebellar peduncles, and longitudinal fasciculi, as indicated by lower fractional anisotropy (FA) and higher mean diffusivity (MD) and radial diffusivity (RD; [49]), although some inconsistencies have been found in younger age groups [90]. Both volumetric and microstructural abnormalities in white matter pathways have been shown to correlate with neurocognitive performance in domains such as processing speed [91], working memory [92], language and reading [42], and mathematics [93] in individuals with PAE. Studies using functional magnetic resonance imaging (fMRI) techniques have also found significant alterations in functional connectivity in FASD thought to reflect underlying white matter dysfunction [46,94,95].

More than 45 years of basic science research has elucidated important mechanisms by which prenatal alcohol exposure damages the developing brain, and the lasting impact of such damage has been illustrated by a large and growing body of neuroimaging data demonstrating abnormalities in brain structure, function, development, and related cognitive deficits [14]. Importantly, cognitive deficits (e.g., in memory and executive functioning) are common in FASD even when IQ is average [96], suggesting that individual neural circuits (e.g., hippocampus, prefrontal cortical circuits) may be the most appropriate targets for intervention rather than global cognition.

## 3. An Overview of Choline and Its Role in Typical Neurodevelopment

Choline is an essential nutrient that is typically grouped with the B vitamins [25]. Without it, cells die by apoptosis [97,98]. It is endogenously produced by the liver but, like other vitamins, must also be consumed in the diet in order to meet one’s daily needs [25]. Choline is found in many foods—the richest sources being egg yolks, meat, fish, poultry, and dairy products. Choline’s functions in the body include serving as a precursor to acetylcholine, phospholipids, and betaine (which is a major methyl donor). Choline is important for cell membrane integrity, lipid transport, transmission of neural impulses, and gene expression [25]. It is required for the production of phosphatidylcholine, sphingomyelin, and plasmalogens present in all cell membranes. In neurons, these phospholipids are necessary for axonal growth and myelination among other developmental processes [26,99]. Furthermore, the ability of cholinergic neurons to produce acetylcholine is directly related to the availability of free choline [100], which is dependent on both endogenous production and dietary intake. During development, choline is metabolically linked to another essential nutrient, folate. Deficiencies in either nutrient lead to neural tube disruptions [101,102,103], and both nutrients are known to be important contributors to normal brain development and cognition [26]. For the interested reader, a comprehensive review of choline metabolism and choline’s mechanisms of action during development are available in [26,98].

In animal models, perinatal choline availability impacts numerous aspects of brain development, especially in the hippocampus. Choline supplementation contributes to increased dendritic arborization in the CA1 region, larger hippocampal cells, and functional changes [104,105,106,107]. Choline is necessary for the development of the hippocampal cholinergic system, and it plays a role in the structure and function of regions essential for memory by contributing to methylation in the hippocampus and prefrontal cortex [26,108,109,110]. Prenatal and postnatal choline supplementation also affect choline acetyltransferase levels in the hippocampus and frontal cortex of rats, which is associated with improved visual-spatial memory functioning [111,112]. 

Critically, the developmental effects of choline are believed to be long lasting and, in some respects, contribute to permanent alterations in brain structure and function. Supplementation during critical early periods of brain development (prenatal and postnatal) induces long-lasting effects on neuroplasticity, including effects that appear to last throughout the lifetime of the organism [113]. Glenn et al. [114] found that prenatal choline supplementation in rats during embryonic days 12–17 led to lower levels of age-related cognitive decline, as well as increased proliferation of hippocampal cells, increased vascular endothelial growth factor, and neurotrophin-3 in the brains of the rats that received choline. The impact of perinatal choline availability on the structural development of the brain has been described as “metabolic imprinting” because of the permanent changes that occur, affecting brain functioning throughout the lifetime [115]. As such, choline holds significant potential as a treatment that may be able to alter the trajectory of neurodevelopment. Although one study has demonstrated an association between choline intake during pregnancy and cognitive outcome in the offspring [116], there is not yet good evidence that supplementation beyond sufficient choline intake during pregnancy results in cognitive gains for the offspring [117]. Nonetheless, choline supplementation during pregnancy may hold value because most pregnant women consume inadequate choline [118], and choline may help optimize aspects of development in high-risk situations. In one clinical trial of pregnant women whose fetuses were at high risk of schizophrenia, Ross et al. [119] found that prenatal and neonatal choline normalized P50 evoked response potentials (ERP)—related to sensory gating and the development of attention deficits in schizophrenia and other conditions. Choline has also been shown to exert a neuroprotective effect in a number of animal models of brain dysfunction, such as prenatal alcohol exposure, aging, Alzheimer’s disease, seizures, stroke, and genetic disorders such as Rett syndrome and Down syndrome [120].

### Mechanisms of Choline’s Action in the Developing Brain

Choline is believed to exert its developmental effects through several mechanisms. First, choline plays a role as a methyl donor for DNA methylation and, thus, it is one of many “environmental” factors capable of regulating gene expression [121], including genes that drive important developmental processes in the brain. Choline also plays a role in histone methylation and has been shown to normalize brain-derived neurotrophic factor (BDNF) concentration following an early insult, with potential downstream benefits for further brain development yet to occur [122]. Deficiencies in choline result in low S-adenosylmethionine concentrations in rodent models, also leading to hypomethylation of DNA. In turn, this altered DNA methylation negatively affects gene transcription, genomic imprinting, and genomic stability [26]. Augmenting choline levels in the face of a deficiency assists in normalizing DNA methylation [123]. Potential epigenetic changes induced by choline are impactful early in development (prenatally), but postnatal gene expression is also relevant for some aspects of continuing neurodevelopment, especially in brain regions characterized by postnatal neurogenesis, such as the hippocampus [124]. In our own studies, we found that 9 months of choline supplementation led to normalized DNA methylation and increased the expression of two genes involved in stress and circadian regulation (POMC and PER2) that are known to be hyper methylated in children with PAE [123]. Further context comes from preclinical studies showing long-term alterations in expression of these genes—particularly in the hippocampus—in animals exposed prenatally to alcohol [125].

In addition to its role in gene expression, choline also serves an important neurodevelopmental role through acting as a precursor to acetylcholine, which functions as both a neurotransmitter and trophic factor in the brain [26]. Choline is known to be readily transported into the brain and incorporated into neurons, resulting in additional acetylcholine synthesis [126,127]. Because there is no strong feedback inhibition for acetylcholine production in neurons, increased free choline in circulation leads directly to increased acetylcholine production in neurons. Cholinergic circuits are critical for memory, in addition to a variety of other functions [110]. In normally developing animals, perinatal supplementation with choline above typical dietary levels enhances performance on measures of cognition, including memory [128,129,130].

A third potential mechanism by which choline affects brain development is through its impact on lipid synthesis. Choline is essential in the formation of major phospholipids involved in cell membranes, including phosphatidylcholine (PtdCho), phosphatidylethanolamine, and sphingomyelin, which are important in maintaining the structure and function of cellular membranes and facilitate axonal growth and myelination [131,132]. During gestation, progenitor cells in the fetal brain require choline to undergo proliferation, migration, and differentiation [132]. PtdCho has been implicated in cell division and growth, and sphingomyelin, a phospholipid derived from PtdCho, is essential for the myelination of axons throughout the central nervous system [133], which begins early in the brainstem (at about 14 weeks gestation) and progresses to thalamic (20 weeks) and cortical axons (35 weeks) in humans [134].

As outlined above, choline clearly has differential impacts on brain development at different stages. During prenatal development, choline directly affects neurogenesis, and it contributes to increased cell proliferation and decreased apoptosis in the hippocampus [108,109]. Later in development, including the postnatal period, choline fosters synaptogenesis, and it continues to impact hippocampal growth [110], which continues at a rapid pace during the first two years [135]. In humans, the hippocampus continues to develop at a slower pace at least into the fourth year of life [136]. In preclinical models, choline supplementation during both “early” (postnatal days 11–20) and “late” (postnatal days 21 to 30) periods attenuates cognitive deficits from PAE and, notably, there is an advantage in early supplementation [137]. In addition to affecting memory systems, there is evidence that choline supplementation improves other aspects of cognitive functioning, including attention [138].

As discussed in a recent paper by Barks et al. [139], there are several caveats regarding the mechanisms of action and timing to consider, as the field potentially moves forward with choline supplementation. For example, although alcohol impacts numerous biological processes (including epigenetic regulation) to cause neurodevelopmental insult, choline likely has a much more discrete developmental impact, and its effectiveness may be discrete as a result. Additionally, the impact of alcohol on the developing brain is likely subject to developmental windows (with different developmental mechanisms being affected at different timepoints), and choline administered later in development may miss those critical windows and/or could theoretically exert other non-targeted effects on brain development. For example, studies of choline supplementation in rat models of hippocampal development have suggested the timing of supplementation is associated with distinct mechanisms affecting hippocampal structure and function when administered in the prenatal or postnatal periods [140]. Lastly, in rodent models of iron deficiency, although the benefits of properly timed choline supplementation have been observed with iron-insufficient animals, there may be evidence of neutral or even negative effects at different timepoints and/or in iron-sufficient animals in the same experimental models [141,142]. Similarly, some studies have found choline supplementation administered throughout the prenatal and early postnatal periods to have negative effects on certain growth factors, such as BDNF [140]. In summary, although choline is clearly important during neurodevelopment, we still have a good deal to learn about how to utilize it as an intervention following neurodevelopmental insult.

## 4. Preclinical Evidence Showing That Choline Augments Development following PAE

### 4.1. Brain Structure and Function

A number of preclinical studies have examined the effects of choline administered before, during, or after ethanol exposure [121]. Idrus et al. [143] administered 1.0, 1.75, or 2.50 g of choline to pregnant, ethanol-exposed rats 2 weeks before conception and throughout lactation. They examined motor and behavioral development in the offspring and found the most severe impairments (delayed eye openings, fewer successes in hindlimb coordination, overactivity) to be in the alcohol-exposed, low choline group. In addition, Glenn et al. [114] conducted a prenatal choline supplementation study in which pregnant rats were administered 5 mg/kg of choline chloride on embryonic days 12 to 17. They measured exploratory behaviors in offspring at 1 and 24 months and found that the younger females that received choline showed increased object exploration behavior, while males showed decreased exploration. Complementing these rodent studies, a large mammal model using prenatal choline supplementation in sheep has also demonstrated significant benefits for brain and eye development following disruption from PAE [144]. The three previous studies examined various areas of development, while Ryan et al. [137] looked at the critical developmental period where choline is most effective in reducing the negative effects of PAE in rats. The rodents received saline or 100 mg/kg per day of choline chloride from postnatal days 11 to 20, 21 to 30, or 11 to 30 and later performed a spatial learning task on postnatal day 45. Choline did not reduce deficits due to ethanol exposure during acquisition, but supplementation during postnatal days 11 to 20 and 21 to 30 decreased deficits in the probe trial. This suggests that the critical developmental period for choline administration is relatively wide in rodents.

Postnatal choline supplementation in rats also contributes to improvement in neurodevelopment [145,146]. In one study, rat pups were exposed to ethanol (or sham) and given choline chloride (or saline vehicle) postnatally. Choline helped mitigate the negative effects of alcohol exposure by normalizing miRNA expression [145]. Otero et al. [146] conducted a similar study in which rat pups received ethanol (or none) on postnatal days 2 to 10, which is equivalent to the third trimester in humans. The pups were also given choline chloride (or saline) on postnatal days 2 to 20. They found that choline reduced hypermethylation caused by alcohol exposure in the hippocampus and prefrontal cortex.

### 4.2. Working Memory and Learning

Rodent models have demonstrated that postnatal choline supplementation can improve the negative effects of PAE on working memory and learning [147,148]. In particular, hippocampus and memory processes dependent on the hippocampus are disproportionate targets of PAE [67,149,150], and choline supplementation offsets some of the learning and memory deficits caused by PAE [151,152]. Schneider and Thomas [147] exposed rats to alcohol from postnatal days 4 to 9 and administered 100 mg/kg of choline chloride or placebo daily from postnatal days 40 to 60 (young adulthood in rats). Choline blunted the negative effects of PAE on spatial working memory but did not mitigate overactivity in the animals. Thomas et al. [152] performed another study examining choline supplementation (postnatal days 2 to 21) in rats prenatally exposed or not exposed to ethanol and its effects on visuospatial discrimination ability. Choline improved task performance in all groups, with larger benefits seen in the ethanol-exposed group. In another study conducted by Waddell and Mooney [148], rat pups were administered ethanol from gestational days 16 to 20 and given 100 mg/kg of choline or saline daily from postnatal days 16 to 30. Rats were randomly assigned to one of four conditions (saline untrained, saline trained, choline untrained, choline trained) and performed a T-maze spatial working memory task. Choline improved the working memory performance of rats in the saline and ethanol-exposed groups. Thus, choline has beneficial effects on memory and learning in rodent models of typical development, as well as prenatal ethanol exposure.

## 5. Human Studies Also Demonstrate Choline Benefits following PAE

### 5.1. Prenatal Choline Supplementation in Humans

In recent years, a handful of human studies have tested prenatal (gestational) choline supplementation as a neurodevelopmental intervention following PAE. Table 1 contains an overview of these studies. To date, a total of three clinical trials with human subjects have been published. In a study conducted in Ukraine, Kable et al. [153] examined the effect of choline supplementation on infant neurophysiology and memory encoding in a sample of 361 pregnant women with moderate/heavy or no prenatal alcohol use. Participants were randomized into three treatment groups: control, multivitamin only, and multivitamin plus choline (750 mg daily). Infant cardiac orienting response, which is an index of learning, was assessed using a habituation/dishabituation paradigm with visual and auditory stimuli at 12-month follow-up. Results indicated a benefit of multivitamin supplementation (with or without choline) on birth outcomes (longer gestation, increased birth weight, length, and head circumference), as well as a positive correlation between multivitamin plus choline supplementation and infant neurophysiological encoding and memory. There were no significant differences in outcome between PAE and non-PAE groups. Changes in plasma choline and choline metabolite during pregnancy were positively related to cardiac orienting responses, suggesting a potential choline effect on some aspects of information processing.

As part of the same study and cohort, Coles et al. [154] explored the effect of choline on infant developmental outcomes at 6 months of age in 367 infants using a common measure of early cognitive and motor development (Bayley Scales of Infant Development). In addition to the expected negative effects of PAE on developmental outcomes (i.e., higher maternal alcohol use associated with worse infant cognitive outcomes), multivitamin supplementation (with or without choline) was associated with higher cognitive functioning. However, there was no effect of choline supplementation (i.e., multivitamin plus choline compared to multivitamin alone) on developmental outcomes. The authors speculated that these findings may have resulted from insensitivity of global infant cognitive testing at this age.

Recently, Jacobson et al. [155] conducted a randomized, double-blind, placebo-controlled clinical trial of prenatal choline supplementation in South Africa. A total of 69 heavy-drinking pregnant women were randomly assigned at mid-pregnancy to 2 mg oral choline per day or placebo, and the treatment continued until delivery. Infants (31 choline, 31 placebo) completed eyeblink conditioning (EBC) and visual recognition memory (i.e., novelty preference) tasks to evaluate the effect of choline supplementation on PAE-impacted early development. Results showed that infants born to mothers treated with choline had superior eyeblink conditioning at 6.5 months of age compared to placebo. Within the choline group, maternal adherence (a proxy for dose received) was strongly related to EBC performance. Similarly, although newborns in both groups were small at birth, infants of choline-treated mothers showed greater “catch-up” in weight and head circumference at 6.5 and 12 months. The incidence of FAS and PFAS was not different between groups. At 12 months, infants in the choline supplementation group showed higher novelty preference scores (a measure of visual recognition memory) compared to those in the placebo group. Importantly, this study used a higher dose of choline supplementation than the previous two studies [153,154]. In a follow-up neuroimaging study of 50 infants in this South African infant cohort, Warton et al. [156] examined regional brain volumes and recognition memory. Infants in the maternal choline group showed normalized volumes relative to the placebo group in 6 out of 12 regions, including the bilateral thalamus and caudate, right putamen, and corpus callosum. Increased volume in these regions correlated with maternal choline supplementation adherence (a proxy for dose received). In addition, increased volume in the right putamen and corpus callosum partially mediated the relationship between treatment group and recognition memory performance found in the original cohort at 12 months of age.

In sum, the results of maternal choline supplementation clinical trials suggest a potential role for choline in ameliorating PAE-related negative growth and behavioral outcomes. It is worth noting that the dose of choline has varied widely across and within preclinical studies and clinical trials, with much higher doses being used in preclinical trials [121]. In the human studies, Jacobson et al. [155] used a higher dose (2 g per day) compared to Kable et al. [153] and Coles et al. [154] (750 mg per day), which may have contributed to differences in outcomes. In addition, procedures for estimating maternal choline intake have also varied across studies, as has the timing of choline supplementation. Differing measures of infant outcomes have also been used, (e.g., physiological measures, such as cardiac orienting response, vs. traditional clinical cognitive measures), which have been shown to have varying degrees of sensitivity in predicting developmental outcomes [121,157]. Finally, the effect of baseline choline intake may contribute to observed outcomes, as choline supplementation may be most effective when there is a choline deficiency [158].

Importantly, even subtle treatment-related effects observed early in life may result in long-lasting benefits later in development, and further longitudinal investigation of the downstream effects of choline supplementation is warranted. Both animal and human studies suggest prenatal choline may have larger effects on neurodevelopment than postnatal choline [121]. However, postnatal studies remain critical because, in the U.S., many alcohol-exposed pregnancies are only identified retrospectively, and nearly half of all pregnancies are unplanned—with rates of unplanned pregnancies even higher in developing countries [159]. In addition, there is a dearth of interventions available for individuals with FASD [8,160]. These issues highlight the importance of postnatal supplementation studies to support the development of children at risk of FASD who were not identified before birth. Furthermore, testing choline’s effects in the U.S., where malnutrition is not common [161], provides critical, complementary information to the studies being conducted in countries such as Ukraine and South Africa, where development is doubly impacted by malnutrition and PAE.

### 5.2. Postnatal Choline Supplementation in Humans

A small number of human studies have tested postnatal choline supplementation in children with histories of PAE. Table 2 contains an overview of these studies. Our group at the University of Minnesota conducted an early double-blind, randomized, placebo-controlled pilot study establishing the safety and tolerability of choline in 20 children with FASD [162]. In this study, 20 children ages 2.5 to 4.9 years with PAE were randomly assigned to two treatment groups for 9 months: 513 mg per day of choline or a placebo. Log sheets and packet counts revealed compliance to be high (82% to 87%). No serious adverse events were reported, although fishy body odor was reported by some participants receiving choline supplementation. Together, the results demonstrated that choline supplementation in young children was safe, feasible, and well tolerated.

A subsequent double-blind, randomized, placebo-controlled trial by our group investigated potential cognitive benefits of postnatal choline supplementation in young children with FASD [163]. A total of 60 children with PAE, ages 2.5 to 5 years, were randomized to receive 513 mg per day of choline or placebo. Again, choline supplementation was feasible and well tolerated, with participants receiving a dose on 88% of enrolled days. Primary and secondary outcomes of this study were global cognitive functioning and a hippocampal-dependent elicited imitation (EI) memory paradigm. Results indicated no effect of choline supplementation on global cognitive functioning. EI performance in this age range reflects development [164], and EI improvement has known implications for future cognitive ability. For example, in one longitudinal study, EI performance at 20 months of age predicted up to 37% of the variance in explicit memory skill at 6 years of age [165]. In the trial, age-related improvements in EI performance were found in the choline group after controlling for immediate recall (thought to reflect attention to the task), with younger participants showing greater improvement than older participants. In mixed-model analyses of growth curves over 9 months, the slope of improvement in EI memory was steeper for the choline group compared to the placebo group (significantly steeper for 2- to 3-year-olds compared to 4- to 5-year-olds). In addition, post hoc analyses of estimated mean daily choline dose received per kilogram of body weight revealed a modest, inverse relationship with EI memory performance (i.e., lower daily dose associated with better performance at 9 months). Importantly, a potential ceiling effect was observed in the EI paradigm and regression toward the mean potentially contributing to this finding. However, the results highlight the need for further study on the cognitive benefits of choline supplementation in young children with FASD and exploration of potential sensitive periods to be targeted with treatment.

In a retrospective analysis of data from this trial [163], Smith et al. [166] examined the role of multiple choline-related single-nucleotide polymorphisms (SNPs) in explaining the cognitive improvement associated with choline supplementation. Results indicated SNPs within the cellular choline transporter gene solute carrier family 44 member 1 (SLC44A1), implicated in transporting choline across plasma and mitochondrial membranes, were significantly associated with EI performance. Performance improvements from choline were dependent on the SLC44A1 allele. These findings emphasize the potential importance of choline-related SNPs in mediating the cognitive effects of choline and highlight the need for future research incorporating genetic analyses.

Recently, we conducted a 4-year follow-up of participants in the original University of Minnesota choline trial [167]. Participants included 31 children (16 who received placebo and 15 who received choline; mean age at follow-up = 8.6 years). Neuropsychological testing included measures of general cognitive functioning, verbal and visual memory, an EI memory paradigm comparable to that used in the initial trial, executive functioning, and ADHD behavioral problems. Participants in the choline supplementation group demonstrated significantly higher non-verbal intelligence, visual-spatial skill, working memory, and verbal memory performance compared to those in the placebo group. In addition, parents rated significantly fewer behavioral symptoms of ADHD in the choline compared to the placebo group.

**Table 2:** Summary of postnatal choline supplementation studies.

Publication	Participants (*n*, Age, Diagnosis)	Choline (Dose, Duration, Form)	Design	Outcome Measures (Cognitive, Behavioral)	Main Findings
Wozniak et al. (2013) [162]	*n*= 20 2.5–4.9 years old with PAE	500 mg choline or placebo 9 months Choline bitartrate	Phase 1 pilot study Double-blind, randomized placebo-controlled trial	Feasibility, adverse effects, tolerability, serum choline levels	Minimal adverse events in choline group, other than a fishy body odor Choline supplementation is feasible and highly tolerable.
Wozniak et al. (2015) [163]	*n*= 60 2.5–5 years old with PAE	513 mg choline or placebo 9 months Choline bitartrate	Double-blind, randomized, placebo-controlled trial.	IQ, elicited imitation (EI) task (i.e., sequential memory)	Improved sequential memory in younger participants (i.e., 2–3 years) who received choline
Nguyen et al. (2016) [168]	*n*= 55 5–10 years old with heavy PAE	29 children had 625 mg choline and 26 children had placebo 6 weeks Glycerophosphocholine	Multisite study Randomized, double-blind, placebo-controlled clinical trial	Memory, executive functioning, attention and hyperactivity	No effects of choline
Wozniak et al. (2020) [167]	*n*= 31; 4 year follow-up after completion of prior Choline study [163] M = 8.6 years old with PAE	Participants had previously received 513 mg of choline 9 months Choline bitartrate	4-year follow-up of randomized, double-blind, placebo-controlled trial	IQ, memory, executive functioning, behavioral and emotional functioning	Choline group: improvements in non-verbal intelligence, visual-spatial skills, working memory, verbal memory, and ADHD symptoms
Smith et al. (2021) [166]	52 children 2–5 years old with an FASD	Children had previously received 500 mg of choline or placebo 9 months Choline bitartrate	Current study was a retrospective analysis utilizing data from the randomized, double-blind trial	Genotyped participants for 384 choline-related single nucleotide polymorphisms (SNPs). Memory and cognition	14-16 SNPs within the SLC44A1 gene largely associated with improved performance on the EI task

CBCL Child Behavior Checklist, EI Elicited Imitation, PAE Prenatal Alcohol Exposure.

Importantly, compared to results of the initial trial [163], which showed moderate group differences in delayed sequential memory, but not overall cognitive skills, group differences at 4-year follow-up were generally larger and more consistent across domains, suggesting that the effects of choline supplementation on neurodevelopment may become more apparent with age [168]. The effects of choline on episodic memory observed at the 4-year follow-up in this study are consistent with findings from two previous studies of prenatal choline supplementation in prenatal alcohol exposure. Kable et al. [153] showed an effect of choline supplementation on an infant measure of visual encoding, and Jacobson et al. [155] reported a choline effect on a visual recognition task. The protracted emergence of choline’s benefits is similar to observed cognitive benefits in other nutritional supplementation trials, including a study of long-chain polyunsaturated fatty acid (LCPUFA) supplements in typically developing infants, in which the beneficial effects on cognitive functioning were not seen initially (18 months of age) but were measurable at 3-year and 6-year assessments [169].

A fourth published human postnatal choline study [168] used a randomized, double-blind, placebo-controlled design to evaluate the effects of choline on cognitive outcomes in older children aged 5 to 10 years old (M = 8.3) with PAE. A total of 55 children were randomized to receive either 625 mg per day of choline (glycerophosphocholine) or placebo (29 choline, 26 placebo) for a total of 6 weeks. Participants in the choline supplementation group did not demonstrate significant differences in primary outcomes measures of memory, executive function, attention, and hyperactivity. The authors note that the null results may be related to a number of factors: the therapeutic window for choline may be early childhood; the duration of supplementation (6 weeks) may have been insufficient; and formulation of choline may play a role.

Together, a handful of postnatal choline supplementation trials have been conducted to date, and the results have suggested positive effects on neurodevelopmental outcomes that may reflect an early developmental window for choline supplementation [168]. Choline supplementation in early childhood is a feasible and well-tolerated intervention with minimal side effects for young children with PAE [162]. The studies summarized here provide evidence that choline supplementation, particularly early in development (i.e., the first few years of life), may confer benefits on hippocampal-dependent memory [163], and additional effects in other cognitive domains, such as non-verbal intelligence, visual-spatial skills, working memory, and improved behavioral symptoms, may become apparent with increased age following early supplementation [167]. Further study is needed to determine optimal timing, dosage, and formulation of choline supplementation, quantify the effects on neurocognitive functioning, explore potential structural and functional changes in the brain, and to better understand genetic moderators of choline’s effects.

## 6. Current Insights into Human Choline Supplementation for Neurodevelopment

Guidelines for the adequate intake (AI) of choline have been established for infants, children, and adults [25] (see Table 3). Adequate choline intake levels for fetuses are less well defined, but it is very clear that pregnant women need additional dietary choline as a result of the added needs of the fetus and the increased prioritization of choline in the fetal circulation at the expense of the maternal circulation [170,171]. Illustrating the importance of choline for early brain development, newborns have free choline concentrations in the range of 35 micromol/L on average—a threefold increase over concentrations seen in adolescents [172,173]. Unfortunately, many adults with “western” diets, such as those in the U.S., consume less than the recommended adequate intake levels of choline; U.S. women consume 278 mg per day, as opposed to the recommended 425 mg per day, or 450 mg while pregnant [174]. Inadequate choline intake is also seen in many other countries around the world [175]. Low choline levels may be seen in diets lacking animal products, such as vegetarian diets. Infants require high levels of choline for brain development, and these levels are impacted by the availability of breastmilk (highest in choline), cow’s milk (moderately lower), or soy-based formula (lower) [25]. Unfortunately, maternal substance abuse compounds the problem of already inadequate nutrition. Alcohol and other drugs interfere with the absorption of nutrients and can significantly lower the availability of important micronutrients, such as choline, to the fetus [176].

These points highlight the fact that fetal development is highly vulnerable to choline deficiency during gestation and that maternal dietary intake is a critical piece of the puzzle [177]. The American Academy of Pediatrics now recommends that women consume additional choline while pregnant [178]. One study found that adding 750 mg of choline per day in the form of phosphatidylcholine to the maternal diet during pregnancy and lactation resulted in no adverse events [117]. Although higher than the 450 mg of choline per day recommended during pregnancy, this amount falls in the range of what could be consumed with a typical diet (1000 to 1500 mg) [170] and is under the tolerable upper intake level of 3500 mg per day [25]. A number of foods contain relatively high concentrations of choline per 100 g of food: beef liver (418 mg), chicken liver (290 mg), eggs (choline is in the yolk) (251 mg), wheat germ (152 mg), bacon (125 mg), dried soybeans (116 mg), pork (103 mg), and ground beef (82 mg) [179]. For comparison, foods lower in choline include beans (27 mg), spinach (22 mg), avocados (14 mg), brown rice (9 mg), oats (7 mg), tomatoes (7 mg), apples (3 mg), and olive oil (<1 mg). A comprehensive database of choline contents in foods is available [180]. Ultimately, for some individuals, reaching 450 to 750 mg of choline per day may be difficult to achieve with diet alone (e.g., with a vegetarian diet or other diet containing low amounts of fish or egg products), and typical prenatal multivitamins often contain only inconsequential amounts of choline. In these cases, targeted choline supplementation may be warranted.

Choline supplementation is relatively easy and is associated with few adverse effects overall, especially at doses below the tolerable upper limit. Very high choline intake can result in hypotension, liver toxicity, sweating, and vomiting, but these symptoms are not seen with dosages under 3500 mg per day [181]. There is literature examining potential negative associations between dietary choline intake and cardiovascular health—mostly in older individuals with cardiovascular disease [182]. In adults, the concentration of one choline metabolite (TMAO) has been associated with acute cardiovascular events in patients undergoing cardiac evaluation or elective coronary angiography [183,184]. However, Loscalzo et al. [185] suggest that “much remains to be done to determine the precise role of TMAO in atherothrombogenesis—whether it has a direct effect on pathogenesis, is an epiphenomenal biomarker, or is a precursor to a more direct effector”. In contrast to these data, a study in hamsters showed an inverse relationship between plasma TMAO and atherosclerosis [186]. Furthermore, fish is a rich source of trimethylamine [187], yet dietary fish intake is associated with decreased risk of cardiovascular disease [188]. One human study demonstrated that consumption of two or more eggs (which are high in choline) increased plasma TMAO concentrations, but there was no association with biomarkers of atherosclerotic plaques [189]. At this point, it is difficult to know if the data from these studies of dietary choline intake and cardiovascular disease have any applicability to studies of choline supplementation for neurodevelopment in very young children without cardiovascular disease.

In our clinical trials, postnatal choline supplementation has proven to be straightforward in children with FASD. In two studies, [162,163] children were able to take 513 mg of choline daily for 9 months with participants consuming a full or partial dose on 88% (IQR = 69–96%) of the days in the study. Choline was no more difficult to take than placebo (days receiving a dose for choline: median: 87%, IQR: 72–93% and placebo: median: 90%, IQR: 69–96% did not differ, *p* = 0.306). Other than fishy odor, there were no adverse events related to choline, and there was no impact on growth, heart rate, or blood pressure. In these studies, using choline bitartrate, we observed free choline concentrations to be 7.33 micromol/L at baseline and 14.77 micromol/L after supplementation (102% increase). Betaine concentrations were 54.5 micromol/L at baseline and 122.23 micromol/L after supplementation (124% increase). Thus, the available serum data confirm, at least for choline bitartrate in the dose administered, that supplementation effectively increases circulating choline levels in children with FASD. At a 513 mg fixed dose, 52% for choline vs. 4% for placebo experienced fishy body odor—a result of excess choline reaching the gut bacteria. Odor prevalence was inversely associated with children’s weight and was universal (100%) in the lightest children. Together, these data led us to transition to a lower, weight-adjusted dose (19 mg/kg per day) for our subsequent studies, currently in the analysis stage (NCT01911299). At this reduced dose, fishy odor has been occurring in only 7.5% of participants.

It is worth noting that a comparison of the baseline serum choline concentration in those with FASD to available normative data [172,173] for the same age range (11.5 compared to 12.8 micromol/L) suggests that children with FASD may have low circulating levels of choline. This is consistent with our earlier finding that children with FASD have inadequate consumption of numerous micronutrients, including choline [190], perhaps due to atypical eating behaviors and taste/texture sensitivities [191]. Therefore, some of the beneficial effects of choline supplementation observed in FASD may be related to bringing these individuals up to a level of sufficiency for choline.

At present, there are minimal data to guide recommendations for the timing of choline supplementation. Based on our understanding of mechanisms of action and on brain development, earlier supplementation (such as prenatal) is advantageous. Furthermore, in our postnatal study of choline supplementation in children with PAE [163], we found that choline had a beneficial effect on hippocampal-dependent memory performance in 2- and 3-year-olds, but a non-significant effect in 4- to 5-year-olds (all from the same cohort), consistent with a possible sensitive period. As discussed earlier, a different study by another research group testing choline in older children with PAE (aged 5 to 10 years) did not demonstrate a positive effect on cognition [168]. At present, the ideal duration of choline supplementation is also unknown. The human prenatal studies in the literature generally supplemented choline through significant portions of gestation. Nguyen et al. [168] supplemented older children for 6 weeks, and Wozniak et al. [162,163] supplemented younger children for 9 months. For context, it is worth noting that many preclinical studies supplemented rodents for 10 to 20 days, which is the equivalent of years in human terms [168]. Our current trial (NCT05108974) is evaluating 3 months of choline vs. 6 months and will also compare the outcomes with our historical 9-month cohort and placebo cohort.

### Formulations and Delivered Choline

It is generally accepted among health professionals that dietary intake of nutrients is the preferred method by which to achieve sufficiency [192]. As previously discussed, egg yolks, meat, fish, poultry, and dairy products are naturally high in choline. Under some circumstances, when higher levels of choline are desired but not easily achieved, supplementation may be necessary. For example, to obtain 750 mg of choline, one would need to consume about five eggs per day.

There are a number of relevant formulations of choline that have been used for supplementation purposes. Each formulation differs in the amount of choline cation delivered, and there may be additional relevant differences in several factors, such as bioavailability, ability to cross the blood–brain barrier, and metabolites (see Table 4). Each form has advantages and disadvantages. For example, choline chloride delivers the highest amount of choline per weight. Glycerophosphocholine is not converted to trimethylamine in the gut and is therefore unlikely to produce fishy body odor with higher doses of choline. It may also be more bioavailable and may cross the blood–brain barrier more easily [193,194].

Choline supplements are commercially available in various formulations from many outlets, including health food stores and online retailers. When purchasing from these sources, inspect the label to determine the actual choline content. For children with sensitivities or specific administration needs, a doctor can prescribe choline supplementation that can be custom compounded by a pharmacy to meet the patient’s individual needs, addressing factors such as flavor, texture, or allergies.

## 7. Conclusions and Future Directions

Fetal alcohol spectrum disorder (FASD) is a highly prevalent, lifelong disability, representing a significant public health burden and associated with a wide range of neurocognitive and behavioral deficits that persist across the lifespan [1,2,3,8,9,21]. Close to five decades of research has led to valuable insights regarding the wide range of effects of PAE on brain structure and function, from genetic factors and cellular mechanisms to brain abnormalities and neurocognitive impairment [14,17,27,28]. Continued global increase in alcohol consumption [6,7], under-diagnosis and misdiagnosis [10,11], and a paucity of available treatments [22,23] highlight the crucial need for novel intervention for individuals affected by PAE. Furthermore, nearly half of all pregnancies are unplanned, and many children with PAE/FASD are only identified postnatally as a result of child protection proceedings, foster-care and adoption screening, state-supported early developmental screenings, and pediatric evaluations, pointing to the urgent need for both prenatal and postnatal interventions with the potential to improve long-term neurocognitive outcomes in this vulnerable population.

Several decades of preclinical research has suggested the essential nutrient, choline, may offset many of the deleterious effects of PAE on brain development and cognitive function, and an emerging line of recent research has investigated potential developmental benefits of choline supplementation in the prenatal and postnatal periods [121]. Studies conducted to date using both animal models and humans suggest prenatal (i.e., gestational) choline supplementation may have larger beneficial effects on neurodevelopment than postnatal choline [121], and the results of postnatal choline supplementation trials in children with PAE similarly suggest earlier supplementation is most effective, possibly reflecting the considerable neurological growth that occurs in the prenatal and early postnatal periods [195,196]. However, the results have varied across studies, likely reflecting, in part, methodological differences (e.g., measurement of cognitive outcomes, duration and dose of choline supplementation), pointing to the need for further investigation. The handful of postnatal choline supplementation trials conducted to date have provided valuable insights into the neuroprotective role of choline in young children with FASD, suggesting that postnatal supplementation is feasible and tolerable and improves neurocognitive function (e.g., hippocampal-dependent memory functioning, aspects of non-verbal and visual-spatial processing, working memory, and behavioral symptoms). Critically, as our existing data show, many beneficial neurocognitive effects are not easily detectable in the short term and may not emerge until later in development for children supplemented at a young age [167]. This may suggest that the beneficial effects of choline supplementation are additive and may compound with age, and there may be a relatively narrow developmental window for postnatal supplementation [168]. Emerging evidence also suggests genetic factors (e.g., choline-related single-nucleotide polymorphisms) may partially mediate neuroprotective effects in this population [166], pointing to the need for further study.

Research conducted to date highlights important avenues for future investigation to further elucidate mechanisms by which choline supplementation in the prenatal and postnatal periods may improve long-term neurodevelopmental outcomes for individuals affected by PAE. Future studies might evaluate the effects of different doses/duration of choline supplementation on specific biological outcomes, such as DNA methylation. Larger sample sizes will allow for a more comprehensive understanding of the role that genetic factors such as single-nucleotide polymorphisms (SNPs, for example SLC44A1, FMO3, BHMT, MTHFD) may play in choline supplementation following PAE. Additional long-term studies are needed to understand the true compounding developmental effects of early rescue and repair by choline supplementation and the role of duration and timing of choline supplementation on neurodevelopmental outcomes. Studies comparing different durations and doses of supplementation will provide valuable insights to maximize the beneficial effects of choline. Preclinical data suggest differential cognitive effects depending on the timing of choline supplementation (e.g., improvement in hippocampal-dependent memory when administered in the early postnatal period, improvement on prefrontal cortex function and working memory when administered during adolescence), and future studies may examine these relationships in clinical trials. Ongoing multidisciplinary collaboration and large-scale, multi-site research initiatives could accelerate research on choline supplementation and provide additional opportunities for investigating potential moderators of supplementation effects in combination with other interventions. Given that many children with PAE/FASD show abnormal eating behaviors [191], future studies may also evaluate dietary interventions and alternative forms of choline supplementation for use with young children. Lastly, neuroimaging studies will continue to elucidate the long-term outcomes on brain structure, complementing studies of functional cognitive outcome.

Going forward, two randomized, double-blind, controlled choline supplementation trials are underway aiming to address several of these important questions. One continuing study, conducted by investigators at Wayne State University with collaborators at the University of Cape Town and Columbia University [156], will next examine gestational choline supplementation in heavy-drinking pregnant women from a rural community in South Africa (NCT04395196). This randomized, double-blind, placebo-controlled study will build on previous work to examine the effectiveness of high-dose maternal choline supplementation (2 g choline bitartrate vs. placebo) on primary outcomes of infant recognition memory and postnatal growth (i.e., weight and head circumference). Secondary outcomes include infant eyeblink conditioning (a basic learning paradigm), postnatal growth (i.e., length), and information processing speed. Additional aims of this study are to evaluate factors associated with compliance and non-compliance and the relationship of maternal dietary choline intake and nutritional status with supplementation effectiveness. An ongoing U.S. study by our group at the University of Minnesota, the fourth in a series of randomized, double-blind controlled trials, investigates postnatal choline supplementation in children with PAE aged 2 to 5 years old (under 6 years old) (NCT05108974). Novel to this study is the examination of multiple cumulative choline supplementation levels and a weight-adjusted dose. Over the course of the 9-month trial, participants are being randomly assigned to receive either 3 months of daily choline (19 mg/kg choline bitartrate) with 6 months of placebo, or 6 months of daily choline (19 mg/kg choline bitartrate) with 3 months of placebo. Primary and secondary outcomes will mirror those used in our previous studies to allow for direct comparison of these novel treatment protocols with our historical 9-month choline and placebo groups across development. The outcomes will include an elicited imitation memory paradigm, cognitive function (IQ), memory and executive functioning, and behavior. A transition to a weight-adjusted dose was prompted by findings from our initial choline supplementation trial indicating an inverse relationship between estimated daily choline intake and memory performance [163], suggesting that a higher dose of choline supplementation is not necessarily superior. This study will provide valuable insights into the necessary cumulative dose of choline associated with measurable cognitive improvement, as well as the long-term effects of cumulative dose across child development.

## Figures and Tables

**Table 1:** Summary of prenatal choline supplementation studies.

Publication	Participants: (*n*, Age, Diagnosis)	Choline Supplementation (Type, Dose, and Form)	Study Design	Outcome Measures	Main Findings
Coles et al., 2015 [154]	*n* = 614 pregnant women (301 consumed alcohol, 313 non-drinking) *n* = 367 children aged 6 months	750 mg choline + multi-vitamin vs. multi-vitamin vs. control 1st prenatal visit - birth	Randomized controlled trial	Cognitive and psychomotor development, orientation/engagement, emotional regulation, motor quality, and total behavior quality.	Higher cognitive development scores in multi-vitamin group, no effect of choline
Kable et al., 2015 [153]	*n* =119 alcohol exposed infants, *n* = 136 controls	750 mg choline + multi-vitamin vs. multi-vitamin vs. control 1st prenatal visit—birth	Randomized controlled trial	Cardiac orienting response to visual and auditory stimuli	Improved cardiac orienting response after choline supplementation Prenatal choline metabolite levels predicted cardiac orienting outcomes
Jacobson et al., 2018 [155]	*n* = 69 heavy drinking pregnant women 62 infants (31 choline, 31 placebo)	2 g of choline bitartrate or placebo mid-pregnancy—birth	Randomized, double-blind controlled trial	Infant growth, recognition memory Eyeblink conditioning (EBC) at 6.5 months; somatic growth at 6.5 and 12 months; recognition memory and processing speed at 6.5 and 12 months	Better EBC task performance and recognition memory in choline group at 12 months Improved infant growth in choline group at 6.5 and 12 months
Warton et al., 2021 [156]	52 infants 52 heavy-drinking women (28 choline; 24 placebo)	2 g choline or placebo daily mid-pregnancy—birth	Randomized, double-blind (after 12-month assessment), choline intervention	Brain structure, recognition memory	Larger brain volumes in 6/12 regions in choline group, which correlated positively with recognition memory in some regions

**Table 3:** Daily adequate intakes for choline.

Age	Male	Female	Pregnancy	Lactation
<=6 months	125 mg	125 mg		
7–12 months	150 mg	150 mg		
1–3 years	200 mg	200 mg		
4–8 years	250 mg	250 mg		
9–13 years	375 mg	375 mg		
14–18 years	550 mg	400 mg	450 mg	550 mg
>18 years	550 mg	425 mg	450 mg	550 mg

NOTE: Table adapted from Food & Nutrition Board, Dietary Reference Intakes [177] and “What we eat in America” [174].

**Table 4:** Choline formulations.

Formulation	% Delivered by Weight	Example	Notes
Choline bitartrate	41.1%	1000 mg delivers 411 mg of choline	GRAS, USP monograph
Choline chloride	74.6%	1000 mg delivers 746 mg of choline	GRAS, USP monograph
Choline citrate	35.3%	1000 mg delivers 350 mg of choline	
Citicholine or CDP-choline	21.3%	1000 mg delivers 180 mg of choline	USP monograph
Glycerophosphocholine (Alpha-GPC)	40.5%	1000 mg delivers 400 mg of choline	Liquid formulations available, % choline variable in these
Phosphatidylcholine	13%	1000 mg delivers 130 mg of choline	Mixture; % choline variable
Lecithin	2–3%	1000 mg delivers 20 mg - 30 mg of choline	Poor source of choline

Notes: Phosphatidylcholine is the predominant choline source in lecithin. Choline content varies significantly for both of these sources [194]. Generally recognized as safe (GRAS) means the FDA has evaluated information, and considers that ingredient to be safe based on expert review. The United States Pharmacopeia (USP) sets standards for quality and purity of various drug and non-drug substances. When a product is labeled USP, it implies a higher standard of quality.

## Data Availability

Data described in this review paper are available from the authors of the cited papers.
